# Construction of a Necroptosis-Associated Long Non-Coding RNA Signature to Predict Prognosis and Immune Response in Hepatocellular Carcinoma

**DOI:** 10.3389/fmolb.2022.937979

**Published:** 2022-07-13

**Authors:** Wenjuan Wang, Yingquan Ye, Xuede Zhang, Xiaojuan Ye, Chaohui Liu, Lingling Bao

**Affiliations:** ^1^ Department of Hematology and Oncology, Beilun District People’s Hospital, Ningbo, China; ^2^ Oncology Department of Integrated Traditional Chinese and Western Medicine, the First Affiliated Hospital of Anhui Medical University, Hefei, China

**Keywords:** necroptosis, lncRNA, hepatocellular carcinoma, immune infiltration, prognosis

## Abstract

**Background:** Necroptosis is a form of programmed cell death, and studies have shown that long non-coding RNA molecules (lncRNAs) can regulate the process of necroptosis in various cancers. We sought to screen lncRNAs associated with necroptosis to predict prognosis and tumor immune infiltration status in patients with hepatocellular carcinoma (HCC).

**Methods:** Transcriptomic data from HCC tumor samples and normal tissues were extracted from The Cancer Genome Atlas database. Necroptosis-associated lncRNAs were obtained by co-expression analysis. Necroptosis-associated lncRNAs were then screened by Cox regression and least absolute shrinkage and selection operator methods to construct a risk model for HCC. The models were also validated and evaluated by Kaplan-Meier analysis, univariate and multivariate Cox regression, and time-dependent receiver operating characteristic (ROC) curves. In addition, Gene Ontology, Kyoto Encyclopedia of Genes and Genomes enrichment, gene set enrichment, principal component, immune correlation, and drug sensitivity analyses were applied to assess model risk groups. To further differentiate the immune microenvironment of different HCC subtypes, the entire dataset was divided into three clusters, based on necroptosis-associated lncRNAs, and a series of analyses performed.

**Results:** We constructed a model comprising four necroptosis-associated lncRNAs: POLH-AS1, DUXAP8, AC131009.1, and TMCC1-AS1. Overall survival (OS) duration was significantly longer in patients classified as low-risk than those who were high-risk, according to our model. Univariate and multivariate Cox regression analyses further confirmed risk score stability. The analyzed models had area under the ROC curve values of 0.786, 0.713, and 0.639 for prediction of 1-, 3-, and 5-year OS, respectively, and risk score was significantly associated with immune cell infiltration and ESTIMATE score. In addition, differences between high and low-risk groups in predicted half-maximal inhibitory concentration values for some targeted and chemical drugs, providing a potential basis for selection of treatment approach. Finally, cluster analysis facilitated more refined differentiation of the immune microenvironment in patients with HCC and may allow prediction of the effectiveness of immune checkpoint inhibitors.

**Conclusions:** This study contributes to understanding of the function of necroptosis-related lncRNAs in predicting the prognosis and immune infiltration status of HCC. The risk model constructed and cluster analysis provide a basis for predicting the prognosis of patients with HCC and to inform the selection of immunotherapeutic strategies.

## Introduction

According to the latest global cancer burden data (Sung et al., 2021), primary liver cancer is the sixth most commonly diagnosed cancer and the third leading cause of cancer death worldwide, with hepatocellular carcinoma (HCC) accounting for 75–85% of cases, and most HCC cases detected at an advanced stage (Singal et al., 2020). Although there are more treatment options for HCC than ever before, even with multidisciplinary treatment strategies, patients with HCC continue to have a poor prognosis, with a 5-year survival rate of approximately 18% (Craig et al., 2020). Although targeted therapies and immunotherapies have recently opened up more options for patients with advanced HCC, the high degree of tumor heterogeneity in HCC limits the overall effectiveness of treatments and the accuracy of predictive prognostic methods (Liu et al., 2016). Therefore, it is particularly important to develop an evaluation model that can improve the efficiency of prognostic prediction for patients with HCC and guide the precise treatment of patients with different disease subtypes.

Necroptosis is a regulated form of cell death mediated by receptor-interacting protein [RIP] kinase 1 (RIPK1), RIPK3, and mixed-spectrum kinase structural domain-like pseudokinase (MLKL), which has a similar mechanism to apoptosis and is morphologically similar to necrosis. Necroptosis is also characterized by the fact that RIPK1 activity can be inhibited by necrostatin-1 (Nec-1) (Degterev et al., 2008; Christofferson and Yuan, 2010). There is growing evidence that necroptosis plays a key role in the regulation of biological processes, including cancer immunity, cancer metastasis, and cancer subtypes (Stoll et al., 2017; Seehawer et al., 2018). As an alternative mode of programmed cell death, necroptosis overcomes apoptosis resistance and may trigger and amplify anti-tumor immunity in cancer therapy (Gong et al., 2019). Further, necroptosis is not only an important cellular process that regulates the evolution of cancer, but is also closely associated with the prognosis of many tumors (Lim et al., 2021; Xin et al., 2022). In HCC, necroptosis driver genes correlate with tumor-infiltrating lymphocyte density, with CD8^+^ T cells clustering in tumors with higher expression of necroptosis-related genes (Nicolè et al., 2022). In addition, targeting RIPK3 signaling, a core factor of necroptosis, is a potential therapeutic strategy to prevent hepatocarcinogenesis (Kondylis and Pasparakis, 2019).

Long noncoding RNAs (lncRNAs) are non-coding RNAs of > 200 nucleotides, which have advantages as cancer biomarkers for diagnosis and prognosis, including dynamic monitoring at different stages of disease, better reflecting disease characteristics (Yan et al., 2015). In addition, lncRNAs can regulate gene expression during different transcriptional stages and epigenetic processes (Fatica and Bozzoni, 2014), and promote tumor inflammation and help malignant tumors to evade immune destruction (Dragomir et al., 2020). LncRNAs differentially expressed in HCC are mainly enriched in biological functions such as tumor metastasis, metabolic regulation, and immune response (Zhu et al., 2021); however, the application of necroptosis-associated lncRNAs to the prognosis and prediction of the immune microenvironment in patients with HCC has yet to be implemented.

Here, we developed a promising HCC model, based on necroptosis-associated lncRNAs, that can be used to predict prognosis and inform the choice of immunotherapy for patients with HCC classified into different clusters.

## Materials and Methods

### Data Collection

Transcriptome datasets and clinical information from HCC tumor and normal group samples were downloaded from The Cancer Genome Atlas (TCGA) database (https://cancergenome.nih.gov). Transcriptomic and clinical information data were subsequently collated using Strawberry Perl (version 5.32.1.1), and mRNA and lncRNA data matrices obtained by differentiating between mRNA and lncRNA for downstream analysis. Based on previous reports (Zhao et al., 2021), 67 necroptosis-related genes were identified for analysis in this study (Supplementary Table S1).

### Identification of Necroptosis-Associated LncRNAs

Expression data for 67 necroptosis-related genes were extracted using the R software package “limma”. A necroptosis-related lncRNA expression data set was obtained by co-expression analysis, using a threshold of Pearson correlation coefficient > 0.4 and *p* < 0.001. A co-expression network of lncRNAs and necroptosis-related genes was constructed using the R software package “igraph”. In addition, “limma” was used to merge lncRNA expression and survival data for subsequent analysis. A co-expression network map of necroptosis-associated genes and necroptosis-associated lncRNAs was generated using Cytoscape software (version 3.9.1).

### Differential Analysis of Necroptosis-Related LncRNAs in Hepatocellular Carcinoma

The R software package “limma” was used to identify lncRNAs differentially expressed between HCC tumor and normal tissues (**|**Log_2_ fold change**|** > 1, false discovery rate (FDR) < 0.05, and *p* < 0.05), and differential heat maps and volcano maps were drawn using the “pheatmap” package.

### Establishment of a Necroptosis-Associated Risk Signature

The R packages “survival”, “caret”, “glmnet”, “survminer”, and “timeROC” were used to obtain prognosis-related lncRNAs and construct prognostic models. First, univariate Cox regression analysis was used to screen out lncRNAs associated with survival (*p* < 0.001) for further analysis. The R package “pheatmap” was used to plot an expression heat map of prognosis-associated lncRNAs. In addition, to elucidate the regulatory effect of these lncRNAs on necroptosis-related genes, “ggplot2” and “ggalluvial” were used to generate a prognosis-related lncRNA Sankey diagram. Then, cases were randomly divided into a training risk group and a test risk group at a ratio of 1:1, and lncRNAs significantly associated with prognosis identified from samples in the training risk group by univariate cox analysis. Candidate lncRNAs were screened using least absolute shrinkage and selection operator (LASSO) regression analysis in the “glmnet” R package, to avoid over-fitting. Risk scores were calculated for each case using the following formula (Zhao et al., 2021):

$$\mathrm{Risk}\;\mathrm{score}=\overset{n}{\underset{k=1}{\sum }}\mathrm{coef}\left(\mathrm{lncRN}A^{k}\right)*\mathrm{expr}\left(\mathrm{lncRN}A^{k}\right).$$

The coef (lncRNAn) in the formula is a shortened form of the coefficient of risk model lncRNAs, and expr (lncRNAn) is the expression of these lncRNAs. Patients were classified into high- and low-risk groups based on the median risk score (Hong et al., 2020).

### Validation of the Necroptosis-Associated LncRNA Signature

R software for univariate and multivariate Cox regression analyses were used to assess whether risk scores and clinical characteristics were independent variables, and “survival”, “survminer”, and “timeROC” used to perform time-related receiver operating characteristic (ROC) curve analysis, to assess the prognostic value of the developed necroptosis-related lncRNA signature. Finally, the “survivor” and “survminer” R packages were used to validate the model and plot survival curves for different clinical subgroups, to determine whether the constructed lncRNA signature was applicable to patients with HCC exhibiting different clinicopathological parameters.

To further explore the association between the constructed lncRNA signature and necroptosis, the R packages “limma”, “reshape2”, “ggplot2” and “ggpubr” were used to analyze the relationship between risk scores and mRNA expression levels of necroptosis-related genes.

### Enrichment Analysis

The R software package “limma” was used to identify necroptosis-related genes differentially expressed between HCC tumor and normal tissues (**|**Log_2_ fold change**|** > 1, false discovery rate (FDR) < 0.05, and *p* < 0.05). Differentially expressed genes enriched in different biological functions were evaluated by Gene Ontology (GO) analysis, comprising three parts: cellular component, molecular function, and biological process; the R packages “clusterProfiler”, “org.Hs.eg.db”, “enrichplot”, and “ggplot2” were used for this process. In addition, the Kyoto Encyclopedia of Genes and Genomes (KEGG) was used to analyze the enrichment of differentially expressed necroptosis-related genes in pathways, using the same R packages as applied for GO analysis; *p* < 0.05 was considered to indicate significant functional enrichment.

Gene set enrichment analysis (GSEA) is a computational method based on existing knowledge of the biological significance, function, and location of genes and has been used to build a database containing multiple functional genomes (Subramanian et al., 2005). GSEA enrichment analysis was performed using GSEA software (version 4.2.3) and enrichment graphs were created using the R packages “ggplot2”, “grid”, and “gridExtra”, to analyze the enrichment of KEGG pathways in the high- and low-risk groups.

### Immunological Correlation Analysis

To explore correlations between the necroptosis-associated lncRNA signature and the immune microenvironment and immune checkpoints, the R software packages “scales”, “ggplot2”, “ggtext”, “tidyverse”, and “ggpubr” were first used to draw bubble charts for immune cell and risk score correlation analysis. Then the “GSVA” and “GSEABase” packages were applied to perform single-sample GSEA (ssGSEA), to obtain immune cell and immune-related function scores for each sample, and the “ggpubr” and “reshape2” packages used to draw ssGSEA difference analysis box line graphs.

ESTIMATE is a technique used to estimate the number of infiltrating immune and stromal cells in tumor tissue (Yoshihara et al., 2013). Here, the number of immune and stromal cells in tumor tissue from each case were calculated using the “ESTIMATE” and “limma” packages in R, to obtain immune and stromal scores; the sum of the immune and stromal scores was the ESTIMATE score, which correlates inversely with tumor purity. Subsequently, box plots of stromal cell and immune cell variability in the high- and low-risk groups were plotted using the “ggpubr” package. Finally, the “ggplot2” and “ggpubr” were used to conduct differential immune checkpoint analysis and to draw box plots of immune checkpoint-associated gene expression in the high- and low-risk groups.

### Clinical Therapeutic Drug Sensitivity Analysis

The R packages “pRRophetic” and “ggpubr” were used to draw box plots to analyze the half-maximal inhibitory concentrations (IC50) of different drugs in the high- and low-risk groups, to explore the correlation of risk models with clinical drug treatment (Geeleher et al., 2014).

### Consensus Clustering Analysis

The R package “ConsensusClusterPlus” was used to cluster tumor samples according to the expression of lncRNAs in risk models and to analyze the relationship between different subtypes and disease risk (Wilkerson and Hayes, 2010). Principal component analysis (PCA) was conducted using the R packages “Rtsne” and “ggplot2”, to further investigate the correlations between different HCC subtypes, the immune microenvironment, and drug sensitivity.

## Results

### Necroptosis-Associated LncRNAs in Hepatocellular Carcinoma

**FIGURE 1:**
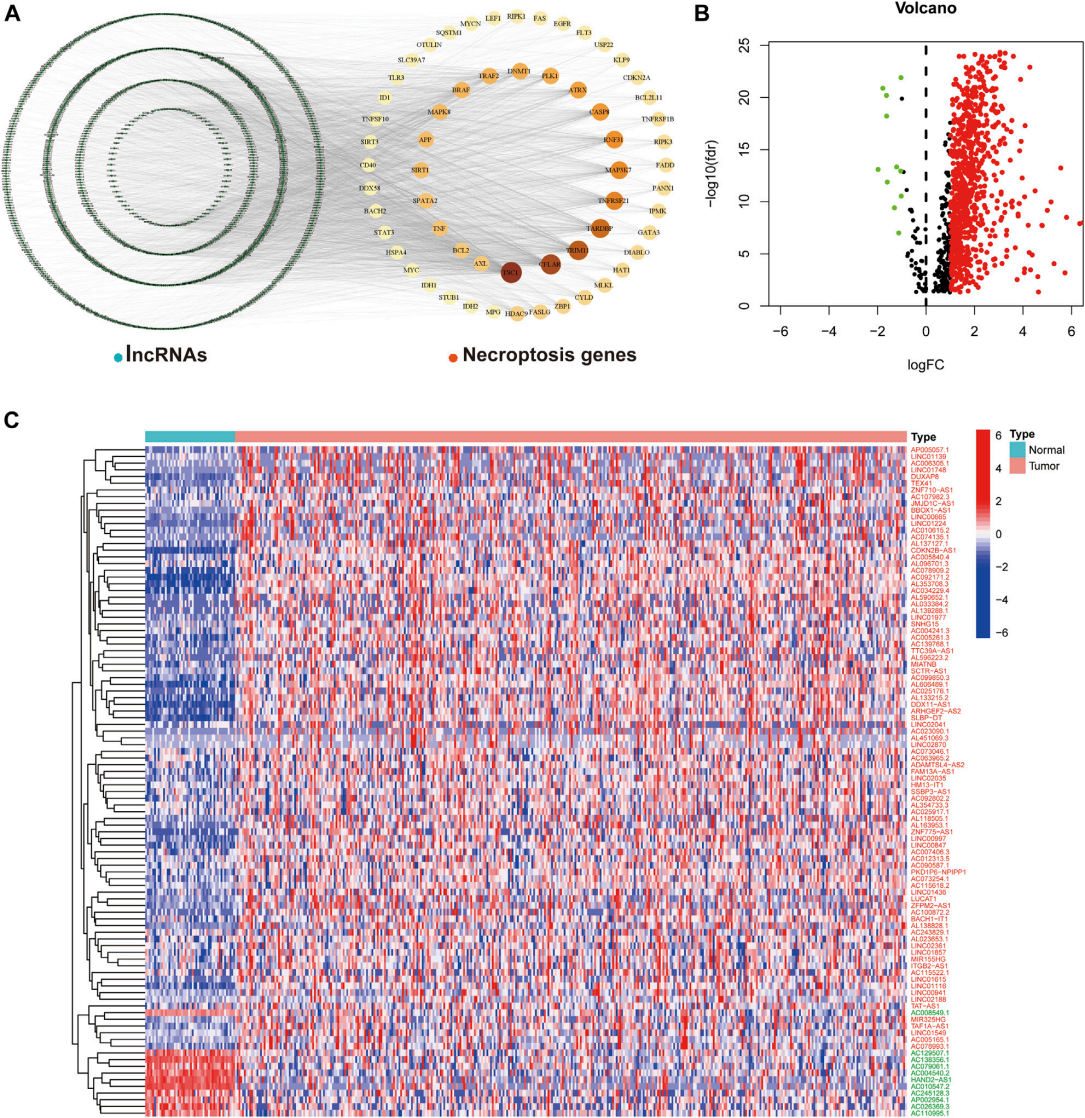
Necroptosis-associated lncRNAs in HCC. **(A)** Co-expression network plot of necroptosis-related genes and lncRNAs. **(B)** The volcano plot of 769 differentially expressed necroptosis-associated lncRNAs. **(C)** Heat map of differentially expressed lncRNAs in normal and tumor samples (Red-labeled lncRNAs represent up-regulated expression in tumor tissues, while the opposite is true in green).

We obtained transcriptome data from 374 HCC tumor samples and 50 normal samples from TCGA. Based on comparison of expression and co-expression of 67 necroptosis-related genes between tumor and normal samples, we obtained 1,015 necroptosis-related lncRNAs (correlation coefficient > 0.4 and *p* < 0.001). This resulted in the construction of a co-expression network of necroptosis-associated genes and lncRNAs (Figure 1A). In addition, a total of 769 necroptosis-associated lncRNAs were differentially expressed in normal and tumor samples (**|**Log_2_ fold change**|** > 1 and *p* < 0.05), of which only 11 were down-regulated and the rest were up-regulated (Figure 1B). The expression patterns of the 11 down-regulated necroptosis-associated lncRNAs and 89 lncRNAs with the highest upregulation coefficients in normal and tumor samples are presented as a heat map in (Figure 1C).

### Model Construction and Validation

**FIGURE 2:**
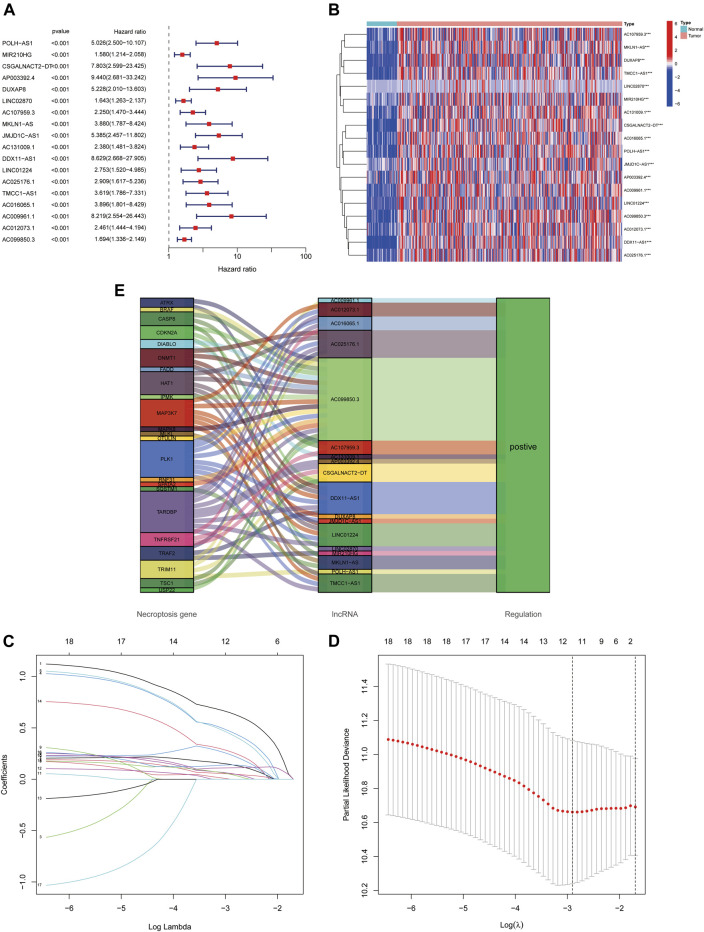
Identification of necroptosis-related lncRNAs prognostic signature. **(A)** The 18 necroptosis-associated lncRNAs extracted by uni-Cox regression analysis. **(B)** Heat map of prognostic lncRNAs expression. **(C)** The Sankey diagram of necroptosis-related lncRNAs and genes. **(D,E)** The coefficient and partial likelihood deviance of the prognostic signature.

Using univariate Cox regression analysis, we found that 18 necroptosis-associated lncRNAs were significantly associated with OS (all *p* < 0.001) as shown in the forest plots and heat maps (Figures 2A,B), all 18 of which were upregulated in HCC, and both positively regulate necroptosis-related genes (Figure 2C). To avoid overfitting the prognostic features, we performed LASSO regression on these lncRNAs (Figures 2D,E), and finally extracted four necroptosis-associated lncRNAs for model construction (Table 1).

**TABLE 1:** Long non-coding RNA signature models associated with necroptosis.

LncRNA	Coef	HR	HR (95%CI)	*p*-value
POLH-AS1	1.306	5.026	2.500–10.107	<0.001
DUXAP8	1.084	5.228	2.010–13.603	<0.001
AC131009.1	0.614	2.380	1.481–3.824	<0.001
TMCC1-AS1	0.831	3.619	1.786–7.331	<0.001

HR, hazard ratio; CI, confidence interval.

**FIGURE 3:**
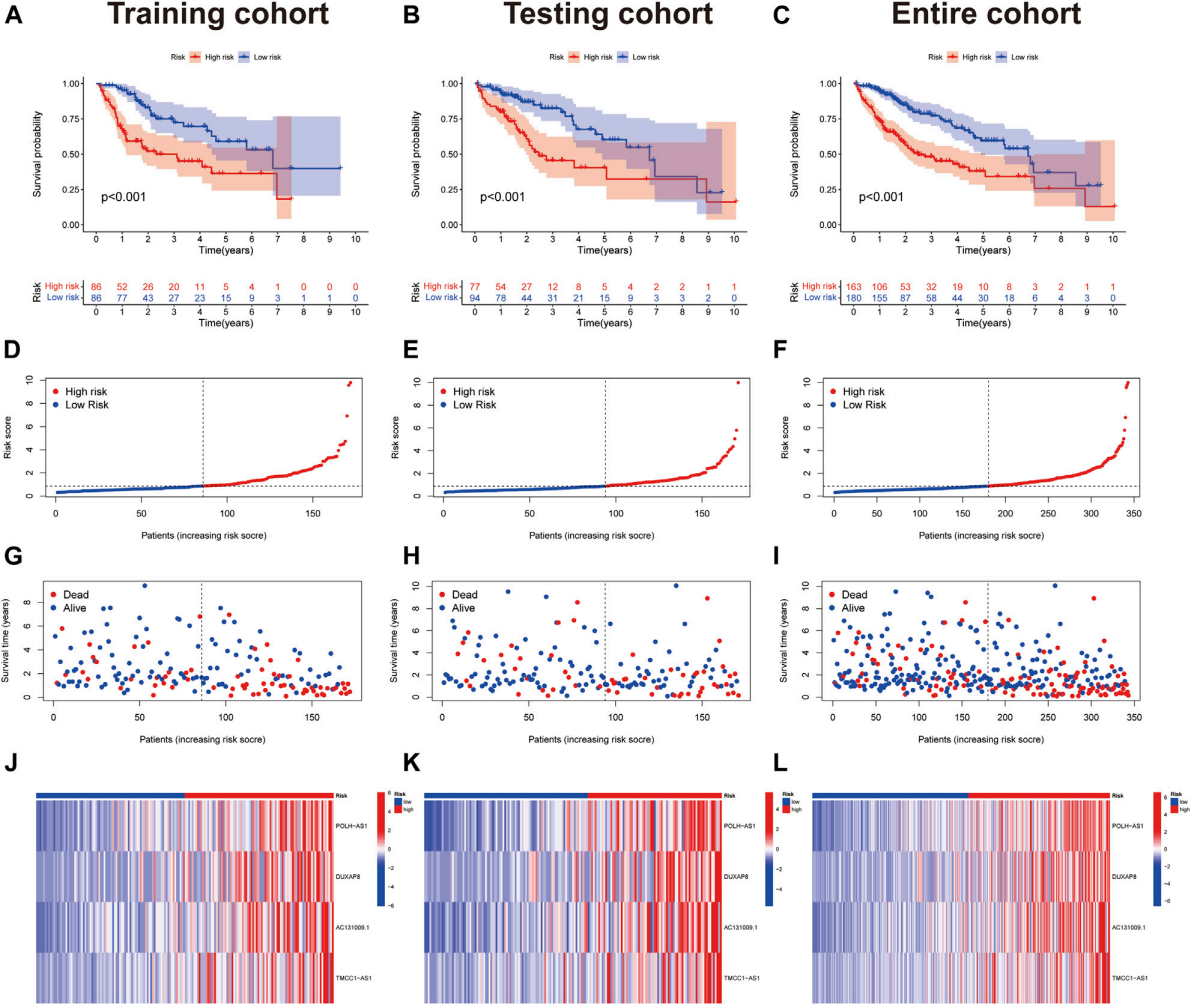
Prognostic value of 4 necroptosis-associated lncRNAs signature. **(A-C)** Kaplan–Meier curve for OS in the train, test, and entire groups, respectively. **(D-F)** Risk score distribution in the train, test, and entire groups, respectively. **(G-I)** Survival time and survival status in the train, test, and entire groups, respectively. **(J-L)** Heat map in the train, test, and entire groups, respectively.

Risk scores were calculated for each case, to assess the survival status, risk score distribution, and survival time of patients in the low- and high-risk groups in the training, test, and total datasets. The results all showed that prognosis was significantly worse in the high-risk group than that in the low-risk group (Figures 3A–I). The heat maps present the expression of the four lncRNAs in the training, test and total datasets (Figure 3J–L).

### Model Assessment

**FIGURE 4:**
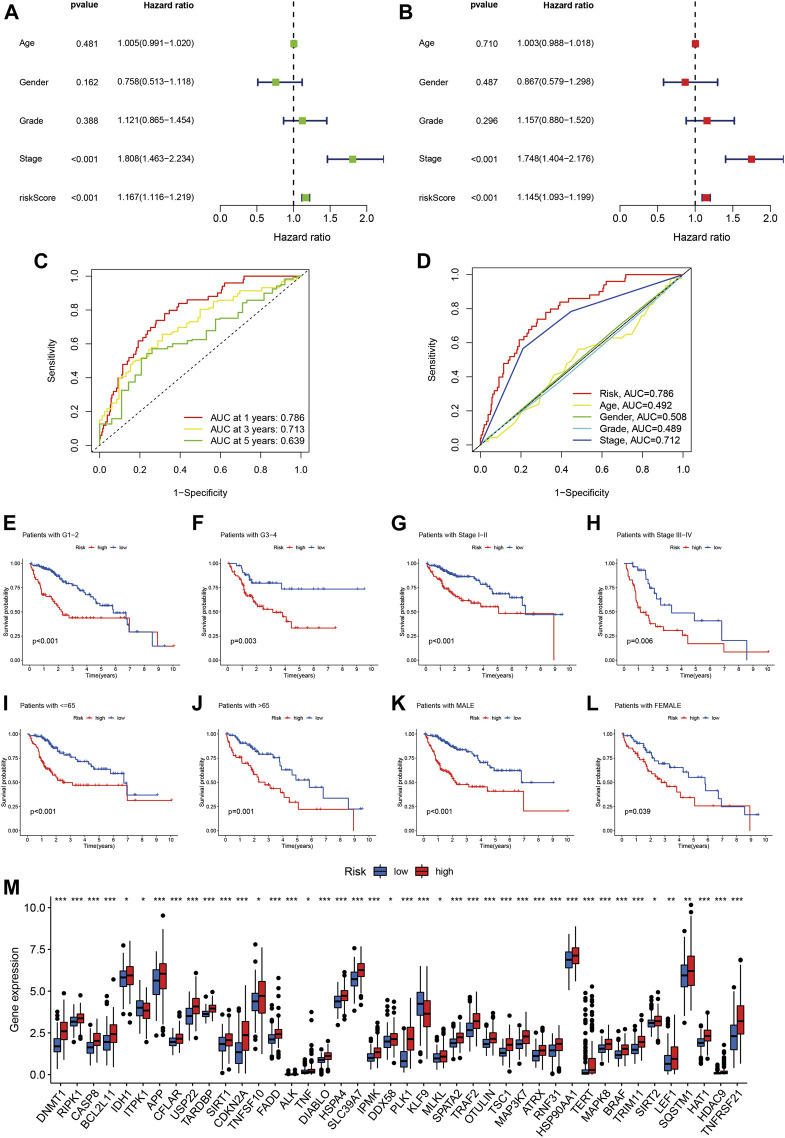
Assessment of the predictive signature. **(A)** Forest plot for univariate Cox regression analysis. **(B)** Forest plot for multivariate Cox regression analysis. **(C)** ROC curve at 1-year, 3-years, and 5-years survival for the predictive signature. **(D)** Comparison of the prediction accuracy of the risk model with grade, TNM stage, age, and gender. **(E-L)** Kaplan–Meier survival curves of low- and high-risk groups sorted by grade, stage, age, and sex. **(M)** Correlation of high- and low-risk groups with the mRNA expression levels of necroptosis-related genes.

Risk score was identified as an independent prognostic factor on both univariate and multivariate Cox regression, with hazard ratio (95% confidence interval) values of 1.167 (1.116–1.219; *p* < 0.001) and 1.145 (1.093–1.199; *p* < 0.001), respectively (Figures 4A,B). Tumor stage was also an independent prognostic indicator.

Time-related ROC curve analysis was used to assess the sensitivity and specificity of the model for prognosis. The results showed that the area under the ROC curve (AUC) values for the risk model were 0.786, 0.713, and 0.639 for prediction of 1-, 3-, and 5-year OS, respectively (Figure 4C), while the AUC value for tumor stage at 1 year was 0.712 (Figure 4D).

We further performed subgroup analysis of HCC patients with different tumor grade, stage, age, and sex, and the results all showed that the prognosis of the high-risk group was significantly worse than that of the low-risk group (Figure 4E-L). Finally, we analyzed the relationship between the risk score and the mRNA expression levels of necroptosis-related genes, and the results showed that 42 necroptosis-related genes were differentially expressed in the high- and low-risk groups, and most of them were highly expressed in the high-risk group (Figure 4M). It is suggested that the constructed risk signature is closely related to necroptosis.

### Gene Ontology, Kyoto Encyclopedia of Genes and Genomes, and Gene Set Enrichment Analysis

**FIGURE 5:**
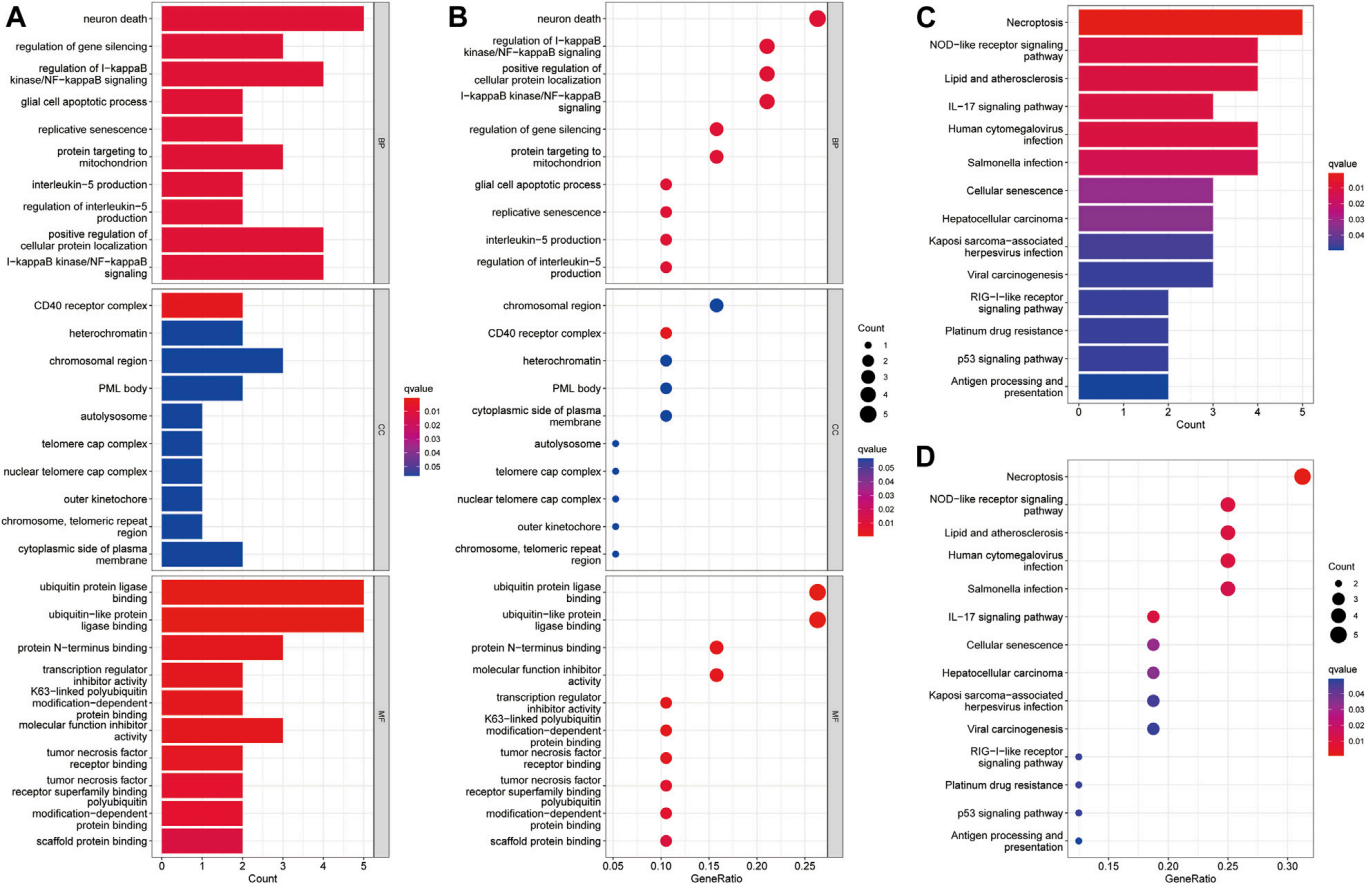
Enrichment analysis of differentially expressed necroptosis genes in HCC. **(A)** Barplot graph for GO enrichment. **(B)** Bubble graph for GO enrichment. **(C)** Barplot graph for KEGG pathways. **(D)** Bubble graph for KEGG pathways. (The longer bar means the more necroptosis genes enriched and the bigger bubble means the more necroptosis genes enriched).

GO analysis suggested that necroptosis-related genes differentially expressed in HCC were mainly enriched for the biological functions, neuron death, ubiquitin protein ligase binding, and ubiquitin-like protein ligase binding (Figures 5A,B). In terms of KEGG pathway analysis, the differentially expressed genes were mainly enriched in necroptosis, NOD-like receptor signaling pathway, lipid and atherosclerosis, and hepatocellular carcinoma (Figures 5C,D).

**FIGURE 6:**
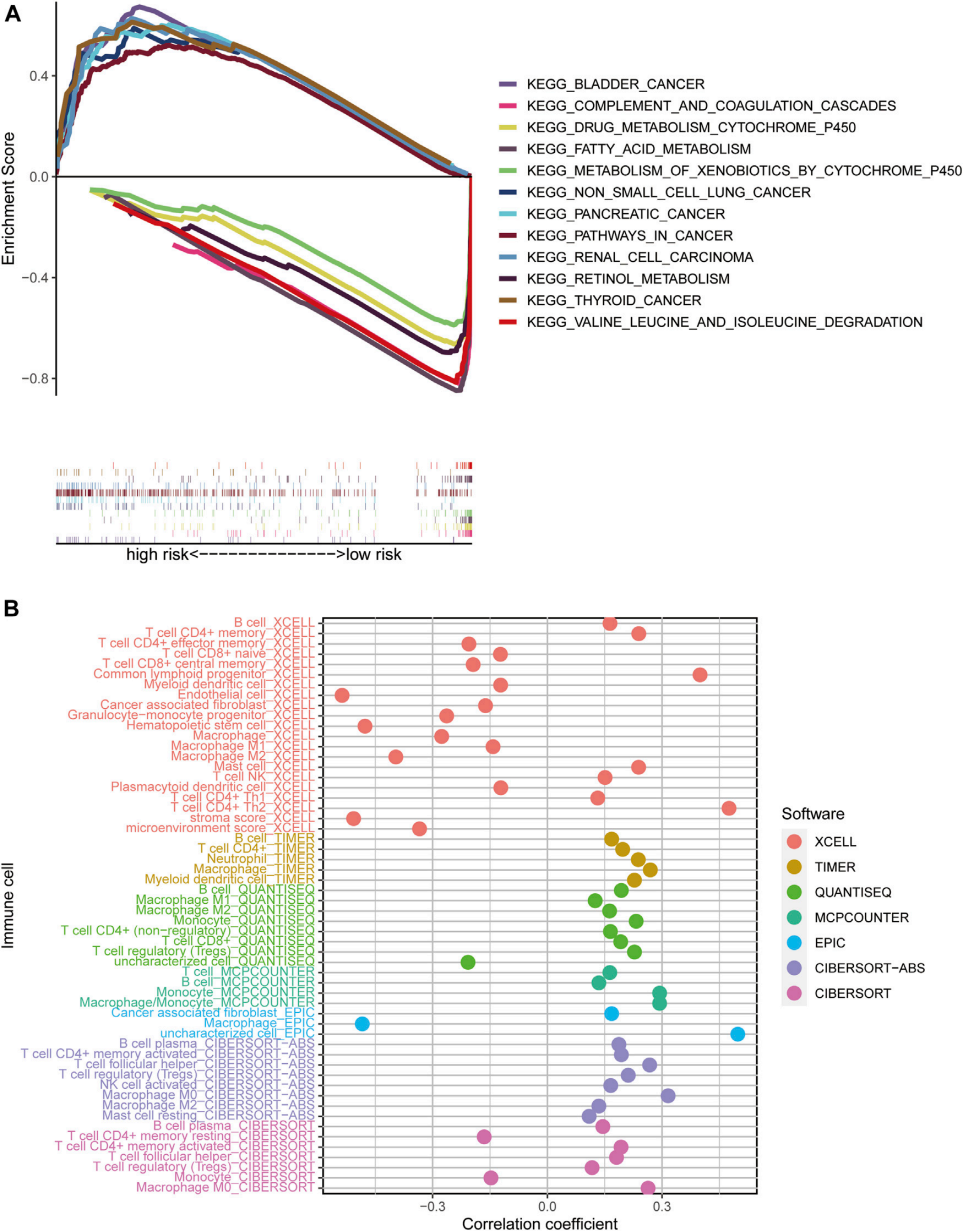
Risk score enrichment pathways and relevance to immune cells. **(A)** GSEA of the 12 pathways significantly enriched in the high- and low-risk groups. **(B)** Bubble plot of correlation coefficients between immune cells and risk scores.

We used GSEA software to investigate the differences in biological function between high- and low-risk groups, according to our model. Pathways enriched in the high-risk group were associated with invasion in a variety of tumors, including bladder cancer, non-small cell lung cancer, renal cell carcinoma, pancreatic cancer, and thyroid cancer, while fatty acid metabolism, retinol metabolism, and cytochrome P450-related metabolic functions were enriched in the low-risk group (all *p* < 0.05; FDR < 0.05) (Figure 6A).

### Investigation of Immunological Factors and Clinical Treatment in Risk Groups

To explore the relationships between different risk groups and the tumor immune microenvironment, we first analyzed correlations between risk scores and immune cell types. As shown in the bubble chart, B cells, CD4^+^ memory T cells, natural killer (NK) T cells, activated CD4^+^ memory T cells, Th1 CD4^+^ T cells, Th2 CD4^+^ T cells, CD8^+^ T cells, and cancer-associated fibroblasts were positively correlated with risk scores, while in the XCELL and EPIC platforms, macrophages were negatively correlated with risk score (all *p* < 0.05) (Figure 6B).

**FIGURE 7:**
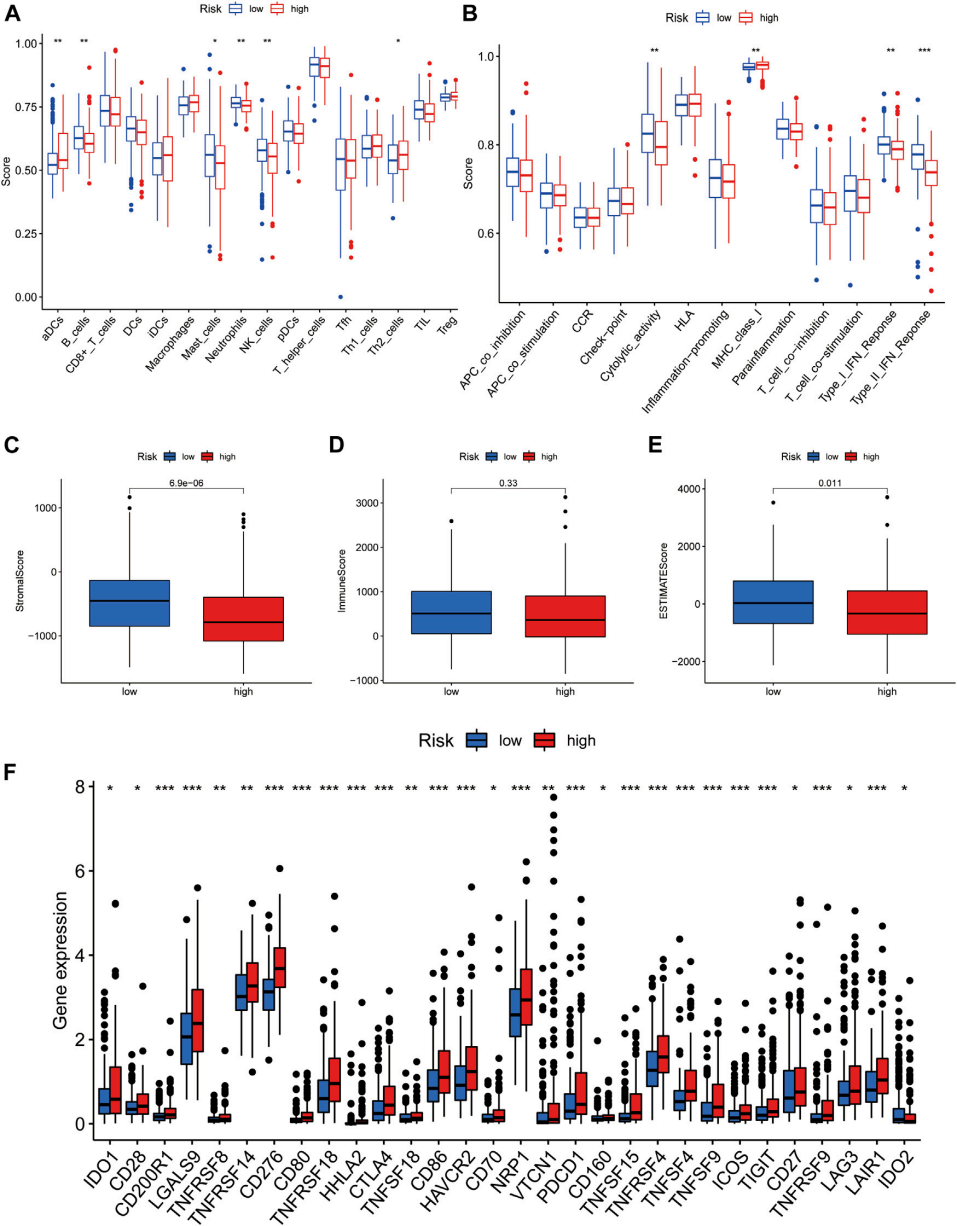
Immunological characteristics of the high- and low-risk groups in the model. **(A)** Comparison of the enrichment scores of immune cells between high- and low-risk groups. **(B)** Comparison of the enrichment scores of immune-related pathways between high and low risk group. **(C)** Correlation of high- and low-risk groups with stromal cell score. **(D)** Correlation of high- and low-risk groups with immune cell score. **(E)** Correlation of high- and low-risk groups with ESTIMATE score. **(F)** Comparison of immune checkpoints in high- and low-risk groups. *p < 0.05, **p < 0.01, and ***p < 0.001.

Box plots showing differential analysis of immune cells and immune-related functions obtained by ssGSEA demonstrated that proportions of B cells, mast cells, neutrophils, and NK cells were significantly lower in the high-risk group than in the low-risk group, while the opposite was true of activated dendritic and Th2 cells (Figure 7A). In terms of immune-related functions, cytolytic activity and type I/II interferon (IFN) response were significantly weaker in the high-risk group than in the low-risk group, while the opposite was true of MHC class I (Figure 7B).

Furthermore, we investigated the relationship between risk groups and ESTIMATE scores. As shown in the box plots (Figures 7C–E), the stromal and ESTIMATE scores of the high-risk group were significantly lower than those of the low-risk group, while the immune score showed a decreasing trend in the high-risk group, but the difference was not significant (*p* = 0.33). These results indicate that the high-risk group may have a lower immune infiltration status. This may partly explain why most immune checkpoints showed better activation status in the high-risk group, to suppress immune function (Figure 7F). These data indicate that our risk model may be useful for immune checkpoint inhibitor selection during clinical decision-making for patients with HCC (Kono et al., 2020).

### Drug Sensitivity in the Risk Groups

**FIGURE 8:**
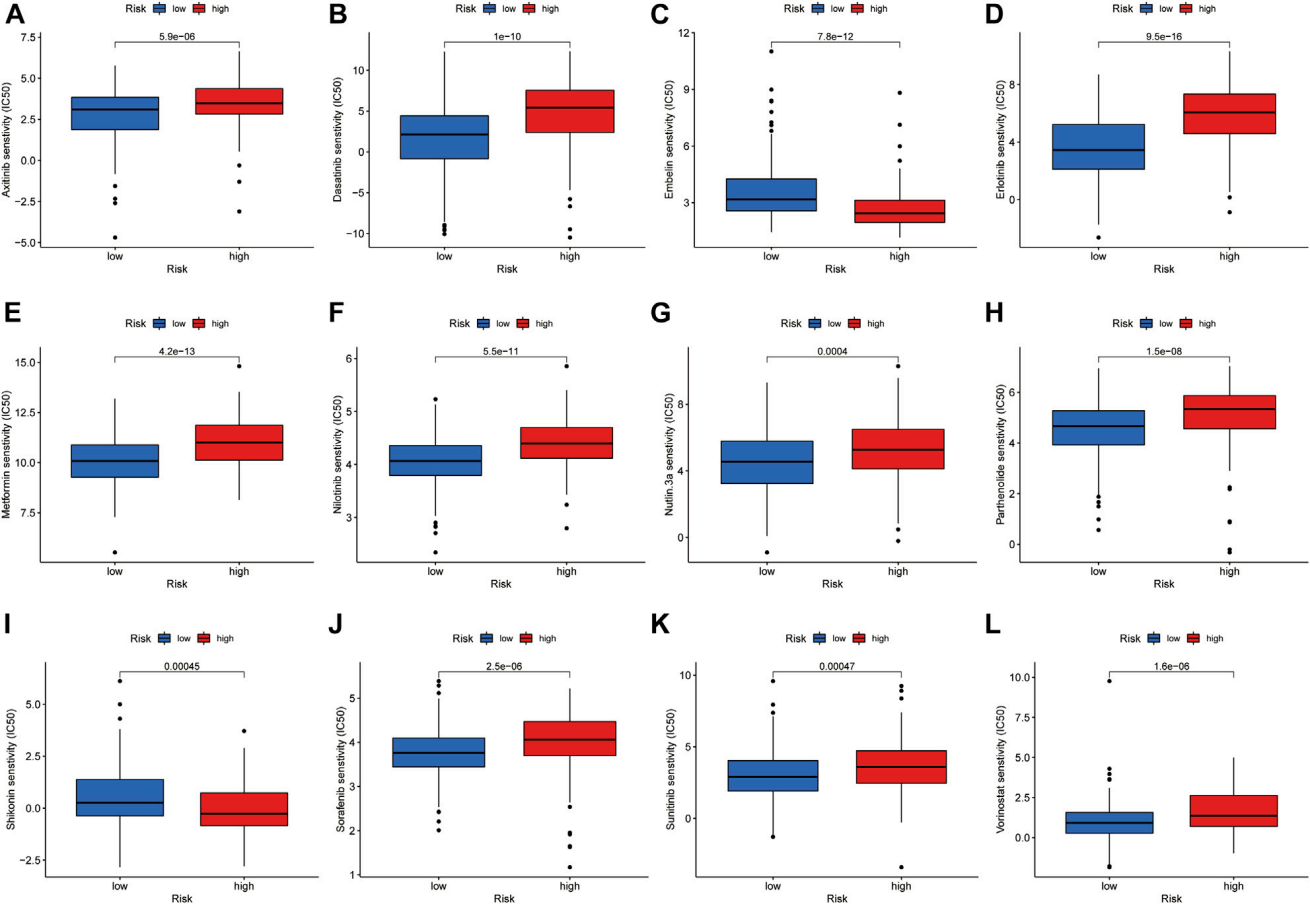
Investigation of drug sensitivity in risk groups. **(A-L)** Comparison of IC50 values for different agents in high- and low-risk groups.

By analyzing the value of the model for assessing drug sensitivity, we found differences in IC50 between the high- and low-risk groups for a variety of chemical and targeted anti-tumor drugs (*p* < 0.001) (Figure 8A–L). Among them, the IC50 values of the tyrosine kinase inhibitors (TKIs), sorafenib, axitinib, sunitinib, erlotinib, nilotinib, and dasatinib, were all higher in the high-risk group than in the low-risk group, suggesting that patients in the high-risk group may be resistant to TKIs.

### Cluster Typing Based on Our Risk Model

**FIGURE 9:**
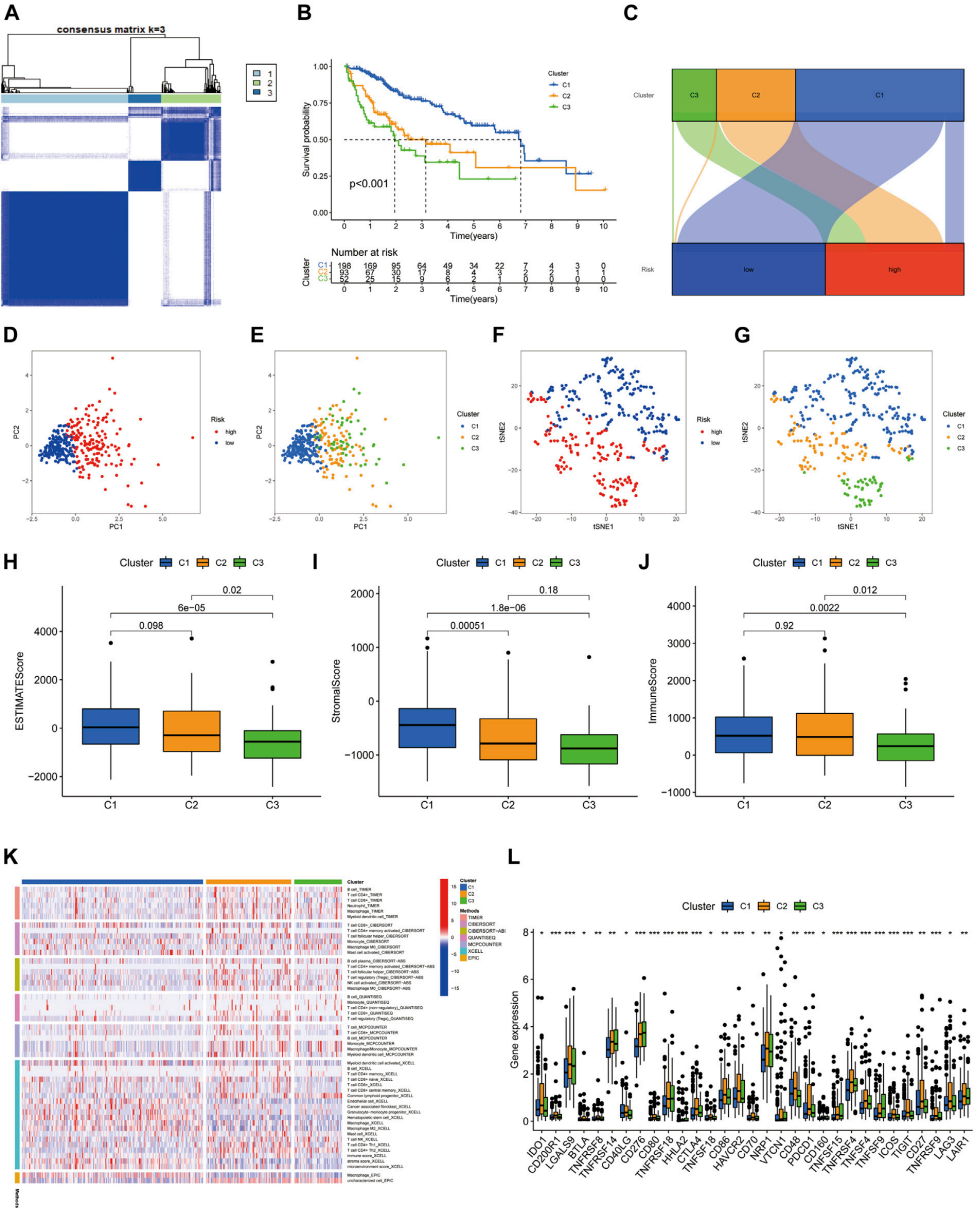
Tumor classification based on four necroptosis-associated lncRNAs. **(A)** HCC patients were divided into three clusters according to the consensus clustering matrix (*k* = 3). **(B)** Kaplan-Meier survival curves for three clusters. **(C)** Sankey diagram of the relationship between the three clusters and risk groups. **(D–G)** Principal component analysis (PCA) and t-distribution random neighbourhood embedding (tSNE) analysis for risk groups and clusters. **(H)** Correlation of stromal cell scores between the three clusters. **(I)** Correlation of immune cell scores between the three clusters. **(J)** Correlation of ESTIMATE scores between the three clusters. **(K)** The heat map of immune cells in three clusters. **(L)** The difference in expression of immune checkpoints in the three clusters. *p < 0.05, **p < 0.01, and ***p < 0.001.

**FIGURE 10:**
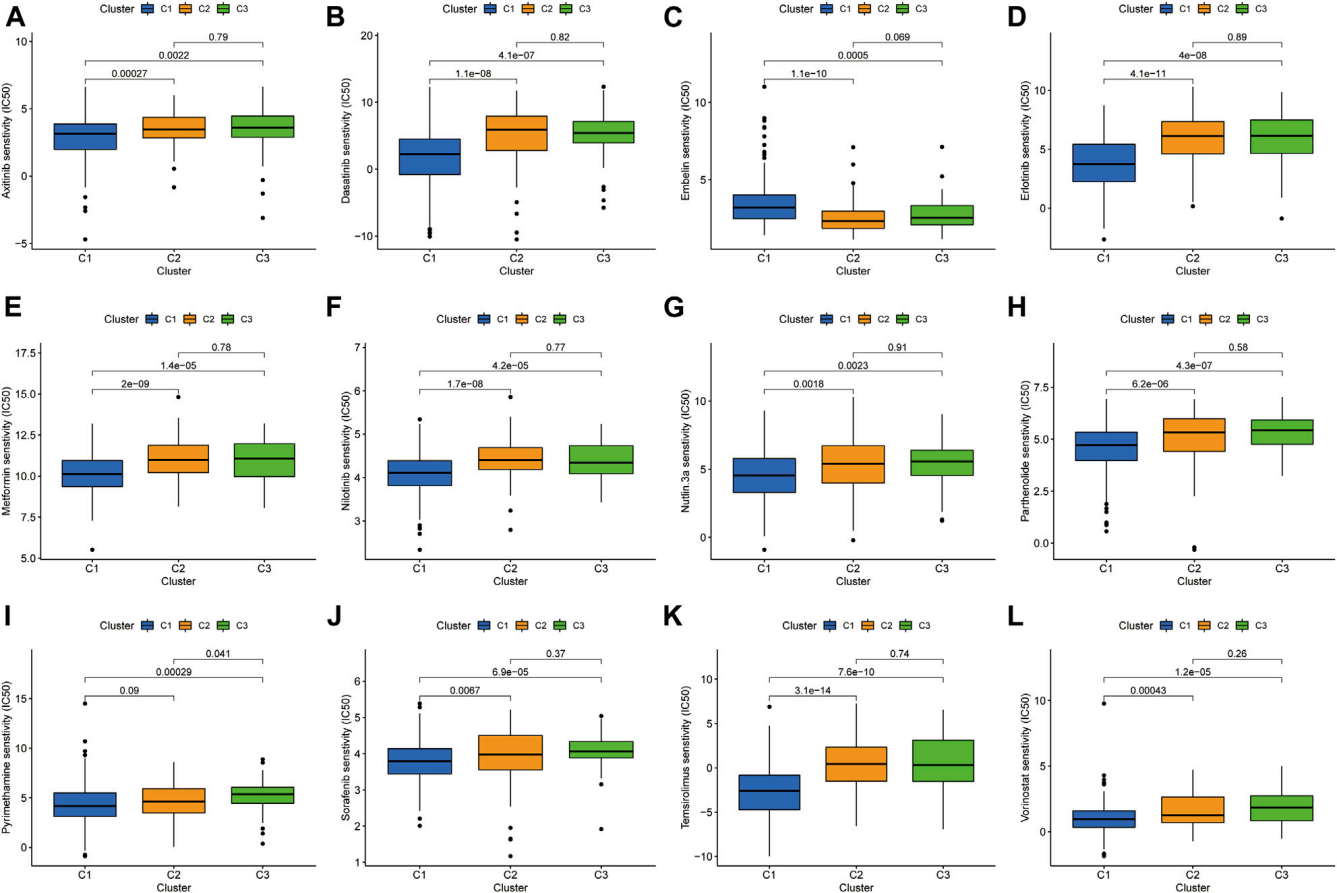
Investigation of drug sensitivity in the three clusters. **(A-L)** Comparison of IC50 values for different agents in three clusters.

Previous studies have confirmed that tumors can be clustered according to their different immune microenvironments, which lead to differences in immunotherapeutic response (Das et al., 2020; DeBerardinis, 2020). We used necroptosis-associated lncRNAs from our risk model to classify patients with HCC into three clusters (Figure 9A). Survival curve analysis suggested that Cluster 1 patients had significantly better survival than those in the other two clusters, with patients in Cluster 3 having the worst survival (*p* < 0.001) (Figure 9B). A Sankey diagram suggested that the majority of patients in Cluster 1 were low-risk, while most of those in Clusters 2 and 3 were high-risk (Figure 9C). Together, these results suggest that clustering can help determine the prognosis of patients with HCC. In addition, both PCA and t-distribution random neighborhood embedding analyses could significantly distinguish between the high- and low-risk groups and the three different clusters (Figures 9D–G). In addition, ESTIMATE analysis was used to investigate differences in the immune microenvironments of clusters. As shown in the box plots (Figures 9H–J), Cluster 1 and 2 samples had higher ESTIMATE and immune scores than those in Cluster 3, while those in Cluster 1 were significantly higher than Clusters 2 and 3 in terms of stromal scores. In addition, we plotted heat maps to further explore the expression of various immune cells in the different subclusters (Figure 9K). Finally, box plots were used to examine the relationships between different clusters and various immune checkpoints, and we observed that the majority of immune checkpoint genes were differentially expressed in different clusters (Figure 9L), suggesting that tumors in the clusters may have different immune microenvironments, which could lead to variations in responses to immunotherapy. Notably, most immune checkpoint molecules were relatively highly expressed in Cluster 2, suggesting that Cluster 2 tumors may have better responses to immune checkpoint inhibitors. Finally, three cluster drug sensitivity analyses (Figure 10A–L) demonstrated that the predicted IC50 values of six TKIs for tumors in Cluster 1 were lower than those in Clusters 2 and 3, suggesting that Cluster 1 tumors may be more sensitive to TKIs. Together, these results suggest that different HCC clusters, differentiated by their expression of four necroptosis-associated lncRNAs, may have variable immunotherapeutic responses, which could provide a basis for individualized and precise treatment using targeted and immune drugs.

## DISCUSSION

Over the past decade, cancer immunotherapy has become a crucial approach to treating patients with cancer (Hegde and Chen, 2020); however, in many cases, immunotherapy response rates are modest. One of the main factors underlying these suboptimal response rates is tumors with a lack, or inadequacy, of tumor T-cell infiltration, referred to as “cold tumors” (Bonaventura et al., 2019), which limits the use of clinical immunotherapy. In contrast, “hot tumors” are immunoinflammatory cancers characterized by high CD8^+^ T cell infiltration and increased IFN-γ signaling (Chen and Mellman, 2017; Liu and Sun, 2021). These tumors are often more sensitive to ICIs (Galon and Bruni, 2019); therefore, clinical differentiation of patient immunophenotypes is important for predicting the prognosis of patients with tumors and the efficacy of immunotherapy.

Necroptosis is established as involved in the tumor immune microenvironment and the anti-tumor immune response, and activation of the necroptotic pathway can enhance tumor cell immunogenicity in the tumor microenvironment (Snyder et al., 2019). In addition, lncRNAs are widely involved in cancer-related cellular pathways and have good predictive power, in terms of tumor prognosis and the tumor microenvironment (Wang et al., 2021; Hong-Bin et al., 2022). Although there have been several studies on the development of new effective lncRNA risk models in HCC (Bu et al., 2022; Liu et al., 2022), the value of necroptosis-related lncRNAs for prognosis prediction and differentiating the tumor immune microenvironment in HCC has not been determined to date.

In this study, we constructed a risk model of necroptosis-associated lncRNAs in HCC. Four necroptosis-associated lncRNAs, POLH-AS1, DUXAP8, AC131009.1, and TMCC1-AS1, were used to predict prognosis and attempt to determine tumor immunophenotype. Among them, DUXAP8 has been reported to mediate the malignant phenotype and chemoresistance of HCC through m6A modification (Liu et al., 2021), and can induce resistance to poly ADP-ribose polymerase (PARP) inhibitors in HCC by upregulating FOXM1 (Hu et al., 2020). Previous reports have shown that POLH-AS1 is associated with ferroptosis (Zhang et al., 2022), suggesting that it plays a role in different ways of programmed cell death. Whereas TMCC1-AS1 is associated with another programmed cell death mechanism, autophagy, in HCC(Deng et al., 2020). Together, these results indicate that the screened lncRNAs participate in the tumor behavior of HCC through multiple pathways. In addition, no studies on the biological functions associated with AC131009.1 have been reported, and its molecular mechanism of action in HCC deserves further exploration.

The model grouped HCC patients into high- and low-risk groups and we performed survival analysis, GSEA, immune microenvironment-related analysis, and IC50 value prediction based on these groupings. The risk model was validated to be prognostic for patients with HCC with different clinicopathological parameters, and was also useful as a guide for treatment with some targeted and chemotherapeutic agents. Of note, sorafenib, a first-generation targeted therapy for HCC, remains the cornerstone drug for patients with advanced HCC (Cheng et al., 2009; Llovet et al., 2018), and our data suggest that low-risk patients will be more sensitive to sorafenib. In addition, erlotinib is a monoclonal antibody that primarily targets EGFR, and a meta-analysis published in 2019 found that bevacizumab in combination with erlotinib is effective and safe for second-line treatment of advanced HCC (He et al., 2019). Sunitinib is an oral multi-target TKI whose main targets are VEGFR and PDGFR, among others. A phase II trial studied sunitinib in combination with transarterial chemoembolization for treatment of advanced HCC, and patients had a median progression-free survival of 8 months and an OS of 14.9 months (Pokuri et al., 2018). Again, our data indicate that low-risk patients will be more sensitive to erlotinib and sunitinib. Overall, the above results suggest that patients in the high-risk group may be resistant to TKIs. In exploration of correlations with the immune microenvironment, although ESTIMATE analysis suggested no significant difference in immune cell scores between the high- and low-risk groups of patients with HCC, those in the low-risk group had superior stromal cell, NK cell, and IFN responses compared with the high-risk group. Immune checkpoints act as immunosuppressive signals, and when overexpressed, they can send a ‘shutdown’ signal to inhibit immune function and thus avoid killing of tumor cells (Brahmer et al., 2012; Pardoll, 2012). In samples assigned to the low-risk group using our model, most immune checkpoint-related genes were expressed at lower levels than in the high-risk group, which may partly explain the better prognosis of the low-risk group and suggest that high-risk patients may benefit more from treatment with immune checkpoint inhibitors.

Previous studies have reported that tumor molecular subtypes are associated with both the tumor immune microenvironment and patient prognosis (Zeng et al., 2019; Zhao et al., 2021). To analyze the differences in immune characteristics of patients with HCC in different subtypes, we classified patients into three clusters according to the expression of risk model lncRNAs. The results suggested that Cluster 1 was mostly classified in the low-risk group in the risk model, which had both superior survival relative to Clusters 2 and 3, and an advantage in stromal cell score. Regarding immune cell score, Clusters 1 and 2 were both better than Cluster 3. Although most samples in Clusters 2 and 3 were classified in the high-risk group, the immune microenvironment characteristics still differed between the two groups, indicating that cluster typing was more accurate in distinguishing immune characteristics than the risk model. Notably, samples in Cluster 2 had higher immune scores and more CD8^+^ T cell infiltration, and most immune checkpoint genes were relatively highly expressed in Cluster 2. Together, these results suggest that Cluster 2 HCC may represent hot tumors that are more sensitive to immune checkpoint inhibitors. Based on the above studies, necroptosis-related lncRNA cluster typing can not only predict the prognosis and immune microenvironment status of patients with HCC, but also provide a basis for predicting the efficacy of immunotherapy. Finally, the predicted IC50 values of sorafenib, axitinib, erlotinib, and dasatinib were lower in Cluster 1 than those of Clusters 2 and 3, in terms of sensitivity to TKIs. Hence, our findings not only facilitate prediction of prognosis and immune characteristics of patients with HCC, but also provide a basis for more accurate individualized treatment strategies.

Although the prediction model generated in this study was validated by different methods, there remain some limitations. First, in retrospective studies, there may be some bias in the included cases. Second, we only used TCGA database data for internal validation, whereas we still need to analyze data from a clinical cohort of patients with HCC for external validation, to test the applicability of the predictive signature. In addition, the mechanism underlying lncRNA association with necroptosis in HCC needs further experimental validation.

## Conclusion

In conclusion, the signature of necroptosis-related lncRNAs identified in this study can effectively predict the prognosis of patients with HCC and provides a basis for understanding the possible mechanism underlying the role of necroptosis-related lncRNAs in HCC, as well as clinical treatment response to TKIs and immune checkpoint inhibitors. Nevertheless, our findings will require further validation in the future.

## Data Availability

The datasets presented in this study can be found in online repositories. The names of the repository/repositories and accession number(s) can be found in the article/Supplementary Material.
