# Supplementary material for: Construction of a Necroptosis-Associated Long Non-Coding RNA Signature to Predict Prognosis and Immune Response in Hepatocellular Carcinoma
Source: Front Mol Biosci. 2022 Jul 13;9:937979. doi: 10.3389/fmolb.2022.937979 (PMC9326067; doi:10.3389/fmolb.2022.937979)
Supplement: Supplementary file 1 [file DataSheet1.ZIP › Supplementary Material/List of Supplementary Materials.docx]

# Supplementary Materials

1. Supplementary Table 1. 67 necroptosis-related genes and source

2. Appendix 1. Co-expression data of necroptosis genes and related lncRNAs

3. Appendix 2. Differential expression data of necroptosis-associated lncRNA in HCC tumor and normal samples

4. Appendix 3. Univariate Cox (uni-Cox) regression analysis data

5. Appendix 4. Expression of the four lncRNAs in the training, test, and whole groups.

6. Appendix 5. Univariate Cox (uni-Cox) and multi-Cox (multi-Cox) regression data for the model

7. Appendix 6. Clinical parameters of HCC patients

8. Appendix 7. Differentially expressed necroptosis gene expression data

9. Appendix 8. Data on the correlation between immune cells and risk scores

10. Appendix 9. ESTIMATE Score analysis data

11. Appendix 10. Cluster analysis data

12. Appendix 11. Differential analysis data of immune cells in different clusters

13. Appendix 12. GO analysis data for differentially expressed necroptosis genes

14. Appendix 13. KEGG analysis data for differentially expressed necroptosis genes
